# Factors predicting home medication management practices among chronically ill older population of selected districts of Nepal

**DOI:** 10.1186/s12877-019-1081-7

**Published:** 2019-02-27

**Authors:** Shakti Shrestha, Ramesh Sharma Poudel, Saroj Pradhan, Aashutosh Adhikari, Arjun Giri, Arjun Poudel

**Affiliations:** 1 Department of Pharmacy, Shree Medical and Technical College, Main Block, Bharatpur-12, Chitwan Nepal; 2 School of Pharmacy, University of Queensland, Brisbane, QLD Australia; 3 Hospital Pharmacy, Chitwan Medical College Teaching Hospital, Bharatpur, Chitwan Nepal; 4 Graduate School of Health, University of Technology Sydney, Sydney, NSW Australia; 5 School of Clinical Sciences, Queensland University of Technology, Brisbane, Australia

**Keywords:** Chronic illness, Older, Medication Management Practice, Medication safety, Nepal

## Abstract

**Background:**

Older population often have multiple and complex needs that are consequently challenged by the presence of polypharmacy, adverse drug reactions and drug-drug interaction. We aimed to determine home medication management practices (MMP) and its associated factors among chronically ill older population of selected districts of Nepal.

**Methods:**

A community based cross-sectional survey was conducted among 386 chronically ill older individuals from selected areas of Nepal between April to September 2016. Appropriateness of MMP was assessed through scores of questions using interview method. Multivariate logistic regression analysis using potential variables from bivariate analysis were used to determine factors affecting MMP.

**Results:**

The overall home MMP was mostly inappropriate (80.1%). Most participants had multiple prescribers for single disease (202, 52.3%) and inappropriate medication storage (188, 48.7%). Though the majority of them had drug administration schedule (378, 97.9%), expired medicines were also used (2, 0.5%). Regression analysis showed less than one year duration of disease (odds ratio [OR] = 3.901, 95% confidence interval [CI] = 1.528 to 9.959, *P* = 0.004), 1–2 years duration of disease (OR = 2.415, 95% CI = 1.210 to 4.821, *P* = 0.012) and smokers (OR = 2.025, 95% CI = 1.036 to 3.956, *P* = 0.039) as the major factors affecting appropriate home MMP.

**Conclusions:**

The home MMP was associated with duration of disease and smoking status among chronically ill older patients living in selected districts of Nepal. Proper counselling and monitoring of such patients might be necessary to improve the practice.

**Electronic supplementary material:**

The online version of this article (10.1186/s12877-019-1081-7) contains supplementary material, which is available to authorized users.

## Background

The demographic shift in the age distribution to an increasingly older population has significant social, health and economic impacts. Health-related issues are challenging among elderly people in terms of prevalence of multiple diseases [1], related medication problems [2] and additional complexity of their changing physiology [3]. Among several medication-related problems, polypharmacy, adverse drug reactions, and the use of potentially inappropriate medications have been commonly reported in the literature [2]. Poor home medication management practice (MMP) also accounts for such problems [4, 5], which is often underestimated in the aged population.

Poor home management of medicines includes poor drug storage practices, lack of medication administration schedule, use of drugs from multiple prescribers, use of discontinued medicines, expired medications or medicines that are no longer needed and the use of over-the-counter medications which are not suitable for their condition [6]. Studies have identified a number of factors associated with these practices including gender, age, confusion between trade and generic names [6], education level [7] and the number of medicines [8]. Particularly with older patients; it has been known that they tend to experience multiple practical problems using their medicines such as difficulty reading and understanding the instruction, handling the packaging, preparation before use, and taking the medicine; all of which have the potential to cause negative impacts on their health [9, 10]. On the other hand, the ageing population is more prone to chronic diseases and is more likely to be prescribed multiple medications [11–13]. Additionally, inappropriate MMP has been linked with a decline in the cognitive functioning of older patients [14]. But several other factors such as wrong storage condition of prescribed medications, omission of medication dose, wrong time and frequency of administration, wrong route of administration, lack of medication administration schedule, use of discontinued or expired medication are often seen as poor home MMP in the older population [6]. It is therefore crucial to understand about the MMP in older population in order to ensure that drugs maintain their potency through appropriate storage along with drug safety by avoiding a mix-up of drugs and minimizing the possibility of overdosing and wastage of resources [15].

Studies on medication management at homes in Australia revealed that most older individuals keep their medicines in the kitchen, around 4% store them in a bathroom and 8.3% store them in multiple locations [6, 16]. Also, 14% of elderly Australians fail to keep their prescription medications in the original container and 9% of them mix more than one prescription medications in the same container [16]. Another study in the similar setting reported that home MMP of chronically ill patients discharged from acute hospital care was also sub-optimal [17]. Similarly, more than half of the patients (52%) discharged from the hospital in the United States were found to have one or more medication discrepancies at home [18]. In another study in the United States it was found that patients with inadequate literacy skills had higher odds (10 to 18 times) of being unable to identify all of their medications as compared to adequately literate skilled patients [19]. A cross-sectional study as a part of a randomized controlled trial conducted in Australia states that community pharmacists and general practitioners have identified number of medication related risk factors such as poor adherence, expired medications, number of prescribers and dispensers, hoarding, multiple storage, no administration schedule, presence of discontinued medication repeats, and confusion between generic and brand names during their home visit [4]. Although the literature on the status of MMP in developing countries is often rare, a study from Uganda reported inappropriate home MMP among 70% of the participants [5]. One of such developing countries is Nepal, which is having an upsurge of older population aged 60 years and above (6.5% in 2001 to 8.1% in 2011) [20]. But there is a paucity of data on MMP among older population from this country. Therefore, we aimed to determine the current status of MMP and its associated factor(s) among chronically ill older population of selected districts of Nepal.

## Method

### Study population and design

A cross-sectional survey was conducted in selected districts of Nepal from April 2016 to September 2016 among chronically ill older population aged ≥60 years who were under medication for chronic illness for at least 6 months, but were able to communicate and take or manage their medication themselves. Those who denied to participate or had impaired communication or were unable to manage or take their medication without assistance, such as those with cognitive impairment, dementia, and psychiatric illness were excluded. A multistage non-random sampling technique was used to collect data. Firstly, a place called Devghat of Tanahun district (Western Region) was purposefully selected because it is a pilgrimage area and high-resident site of older people. Similarly, two districts in its periphery, namely Chitwan (Central Region) and Nawalparasi (Western Region), were selected. The places inside these districts were also selected due to them being close to Devghat, namely Bharatpur and Ratnanagar of Chitwan district, and Gaidakot and Kawasoti of Nawalparasi district. A total sample size of 386 was conveniently taken each from Devghat (*n* = 106), Bharatpur (*n* = 142), Ratnanagar (*n* = 31), Gaidakot (*n* = 67) and Kawasoti (*n* = 40).

### Data collection and measurement tool

The questionnaire on MMP was developed based on previous literatures [4, 5] and any issues of clarity, specificity of variables to be measured and relevance of the contents of the questionnaire in our context were taken into consideration (see Additional file 1). Data was collected using house-to-house survey by Bachelor of pharmacy final year students who were trained on the objectives of this study, medication management assessment techniques, and approach and interviewing techniques for older population. A face-to-face interview was used to collect data. The data collection sheet consisted of questions on demography (age, sex, education, and marital status); illness (types of diseases, duration of diseases); lifestyle (alcohol intake, smoking, tobacco chewing, walk or yoga) and home MMP. The MMP was assessed using seven questions that covered drug administration schedule, drug hoarding practice, use of expired medications, multiple prescribers, route of drug administration, storage condition and medicine duplication. Each correct answer was scored ‘one’. A total score of seven was considered appropriate MMP whereas any score below seven was considered inappropriate practice. Drug hoarding, multiple prescribers, medicine storage and medicine duplication are elaborated below.

#### Drug hoarding

We considered drug hoarding if the participant had their prescription drugs even if they were discontinued.

#### Multiple prescribers

More than one prescribers for the same disease condition only. Face-to-face interview was used to minimize the error on multiple prescribers in cases where patients with multiple diseases had multiple prescribers.

#### Medicine storage

Storage was assessed considering the place of storage, issues with the container of medicines and accessibility from children (if present). Storing in a cool and dry place or refrigerator according to the requirement was appropriate. On contrary storing in bathrooms or kitchens or other humid places or in direct sunlight was inappropriate. Additionally, a single place of storage for all medicines unless otherwise instructed or justifiable was appropriate. However, storing semi-solid dosage forms like creams and ointments in a separate container from other medicines was an appropriate practice. Storage of medicines required to be in a refrigerator would be inappropriate if stored at room temperature or not stored in an appropriate container inside it and not kept separately from foods and other consumable items. Medicines easily accessible to children at places such as tables or low shelf or unlocked places were inappropriate.

#### Medication duplication

Presence of same drug or drugs of the same therapeutic category in two or more medicine brands was defined as medication duplication. It was considered to be present if participants had in hold duplicate medications regardless of whether they were taking them concurrently.

### Statistical analyses

Descriptive statistics were performed for all variables and the association of MMP with other study variables was assessed using the Chi-square test and Mann-Whitney U test where appropriate. Among all the variables, age was the only numeric variable and it did not pass the test of normality (Shapiro-Wilk test *p* < 0.05). Hence, Mann-Whitney U test was used to determine the median difference of age across the two categories of MMP (appropriate and inappropriate). Multivariate logistic regression analysis was performed to determine the factors predicting MMP. A *p*-value of < 0.05 was considered statistically significant. Data were analyzed using IBM-SPSS 20.0 (IBM Corporation, Armonk, NY).

### Ethics

Ethical approval of this study was obtained from the Chitwan Medical College- Institutional Review Committee (CMC-IRC) and verbal informed consent were obtained from each participant. Verbal consent was used due to higher rate of illiteracy among older population of Nepal. Also the study protocol presented no risk or harm to the participants and the method of consenting was approved by the IRC. For those participants who were under the care of family members or care-staff, the consent was first sought from the available next-of-kin or care-staff.

## Results

### Demographic characteristics

The demographic characteristics of the study population have been depicted in Table 1. Age of the participants ranged from 60 to 96 years, with the median age [interquartile range (IQR)] of 69 (11) years. Males were predominant (58.5%) in this study. Most of the participants never received formal education during their lifetime (227, 58.8%) and below a quarter (90, 23.3%) of the participants just received primary education. The majority of the participants were married (255, 66.1%) while 103 (26.7%) were widows/widowers.

**Table 1:** Baseline characteristics of the study participants (*n* = 386)

Characteristics		Categories	n (%)
Demography	^a^Age (years)	60–96	69(11)
	Gender	Male	226(58.5)
		Female	160(41.5)
	Level of education	Never went to school	227(58.8)
		Primary	90(23.3)
		Secondary	38(9.8)
		Higher secondary	24(6.2)
		Graduation and above	7(1.8)
	Marital Status	Unmarried	4(1.0)
		Married	255(66.1)
		Divorced	3(0.8)
		Widowed	103(26.7)
		Separated	21(5.4)
Illness	Types of disease	Hypertension	126(32.6)
		Diabetes	68(17.6)
		Asthma	45(11.7)
		Rheumatoid Arthritis	14(3.6)
		Multiple diseases	75(19.4)
		Others	58(15.0)
	Duration of diseases	More than 6 months	29(7.5)
		More than 1 years	64(16.6)
		More than 2 years	75(19.4)
		More than 3 years	218(56.5)
Lifestyle	Alcohol consumption	Yes	66(17.1)
		No	320(82.9)
	Smoking	Yes	95(24.6)
		No	291(75.4)
	Chewing tobacco	Yes	53(13.7)
		No	333(86.3)
	Go for morning/evening walk or do yoga	Yes	189(49.0)
		No	197(51.0)

^a^Median(IQR) instead of n(%)

### Illness characteristics

Of the 386 participants, the majority were suffering from hypertension (126, 32.6%), followed by diabetes (68, 17.6%) and asthma (45, 11.7%). Fifty-eight (15%) participants had other diseases like gastritis, urinary tract problem, and heart problem. Likewise, 75 (19.4%) participants were suffering from more than one chronic disease. Most of the participants (218, 56.5%) were suffering from chronic diseases for more than three years (Table 1).

### Lifestyle characteristics

The number of participants taking alcohol, cigarette and chewing-tobacco were 66 (17.1%), 95 (24.6%) and 53 (13.7%), respectively. Almost half of the participants did morning or evening walks or did some form of physical exercises (Table 1).

### Home medication management practice

**Fig. 1:**
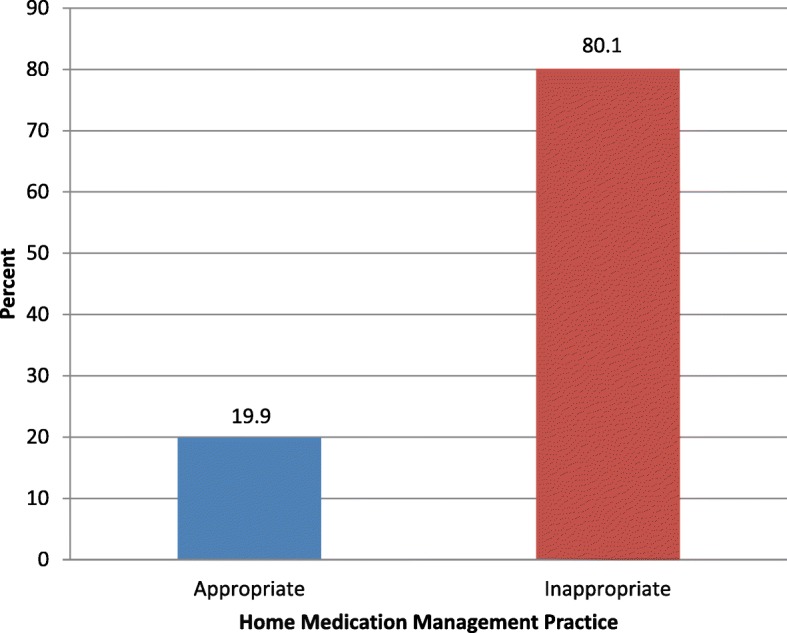
The overall home medication management practice (*N* = 386)

Significant number of participants (378, 97.9%) had drug administration schedule. Two participants (0.2%) used expired medicines. More than half of the participants were also reported to visit more than one prescriber for their disease condition whereas 166 (43%) hoarded drugs for future use. One hundred ninety-eight participants (51.3%) were found to store their medication appropriately. Among those with poor storage conditions, most of them had no specific location for medicines (88, 22.8%) and kept them in inappropriate locations (60, 15.5%) (Table 2). On the basis of seven questions as assessed in Table 2, four-fifths of the participants (309, 80.1%) had inappropriate home MMP (Fig. 1).

**Table 2:** Individual aspect of medication management practices of the participants (*n* = 386)

Characteristics	Categories	n(%)
Have drug administration schedule	Yes	378(97.9)
	No	8(2.1)
Hoard/stock drug	Yes	166(43.0)
	No	220(57.0)
Use expired medicines	Yes	2(0.5)
	No	384(99.5)
Have multiple prescribers for disease condition	Yes	202(52.3)
	No	184(47.7)
Follow correct route/way of drug administration	Yes	380(98.4)
	No	6(1.6)
Have actual medication duplication	Yes	13(3.4)
	No	373(96.6)
Storage condition	Good condition	198(51.3)
	No specific location for medicines	88(22.8)
	Multiple medicines storage area	27(7.0)
	Medicines in wrong container	13(3.4)
	Keep medicines in inappropriate location	60(15.5)

Table 3 demonstrates that there was a statistically significant association of home MMP with the duration of disease at *p* = 0.002. It can be observed that the highest number of participants who had inappropriate MMP (183, 59.2%) and who had appropriate practice (35, 45.5%) were those under medication for more than three years. Also, those who had inappropriate practice were slightly older than those who had appropriate practice (69 years vs 67 years). Inappropriate MMP was high for those participants who never went to school (186/386) though those with graduation level of education also failed to have appropriate MMP (7/386). Similarly, a higher percentage of individuals with inappropriate MMP were seen in those who had alcohol intake habit [55(83.3%)] and were smokers [82(86.3%)] compared to those without alcohol intake habit [11(16.7%)] and who were non-smokers [13(13.7%)].

**Table 3:** Association of baseline characteristics with medication management outcome practice (n = 386)

Independent variables		Medication management practice		*P*-value
		Inappropriate	Appropriate	
^a^Age (years)		69(12)	67(10)	0.176
Gender	Male	179(79.2)	47(20.8)	0.714
	Female	130(81.2)	30(18.8)	
Level of education	Never went to school	186(81.9)	41(18.1)	0.256
	Primary	73(81.1)	17(18.9)	
	Secondary	26(68.4)	12(31.6)	
	Higher secondary	17(70.8)	7(29.2)	
	Graduation and above	7(100)	0	
Marital status	Married	200(78.4)	55(21.6)	0.329
	Single	109(83.2)	22(16.8)	
Types of disease	Asthma	40(88.9)	5(11.1)	0.225
	Diabetes	56(82.4)	12(17.6)	
	Hypertension	93(73.8)	33(26.2)	
	Rheumatic arthritis	10(71.4)	4(28.6)	
	Other disease	49(84.5)	9(15.5)	
	Multiple diseases	61(81.3)	14(18.7)	
Duration of disease	< 1 years	18(62.1)	11(37.9)	0.002*
	1–2 years	46(71.9)	18(28.1)	
	2–3 years	62(82.7)	13(17.3)	
	> 3 years	183(83.9)	35(16.1)	
Alcohol consumption	Yes	55(83.3)	11(16.7)	0.573
	No	254(79.4)	66(20.6)	
Smoking	Yes	82(86.3)	13(13.7)	0.107
	No	227(78.0)	64(22.0)	
Chewing tobacco	Yes	42(79.2)	11(20.8)	1.000
	No	267(80.2)	66(19.8)	
Go for morning/evening walk or do yoga	Yes	149(78.8)	40(21.2)	0.647
	No	160(81.2)	37(18.8)	

The values in the column ‘Appropriate’ and ‘Inappropriate’ of the outcome variable inside the small bracket are the percentage within independent variables and those outside the bracket are the count per total unless otherwise marked in variable. ^a^Median(IQR)& Mann-Whitney U test, ^*^Significant at *P* < 0.05 and where not marked chi-square test was performed

Table 4 shows regression model for predicting appropriate home MMP developed from multivariable logistic regression analysis of explanatory variables (*p* < 0.25 from Table 3), namely age, disease type, duration of disease and smoking status. The model shows that the duration of disease and smoking status are statistically significant predictors of appropriate home MMP. Those with lesser duration of the disease had higher odds of appropriate MMP but the odds were statistically significant only with participants with disease duration of less than one year and 1–2 years when compared with the duration of more than three years. Individuals aged ≥60 years with less than one year duration of disease (*p* = 0.004) and 1–2 years duration of disease (*p* = 0.012) had 3.901 (95% CI 1.528 to 9.959) and 2.415 (95% CI 1.210 to 4.821) times higher odds of appropriate MMP, respectively than those with more than three years duration of disease. Similarly, smokers had 2.025 times lesser odds (95% CI 1.036 to 3.956) of appropriate home MMP than non-smokers at *p* = 0.039.

**Table 4:** Regression model for prediction of appropriate home medication management practice (n = 386)

Variables		Reference category	β (SE)	OR(95% CI)	*P*-value
Duration of disease (*P* = 0.006*)	*< 1 years*	> 3 years	1.361 (0.478)	3.901 (1.528 to 9.959)	0.004*
	*1–2 years*		0.882 (0.353)	2.415 (1.210 to 4.821)	0.012*
	*2–3 years*		0.025 (0.373)	1.025 (0.493 to 2.131)	0.946
Smoking status	Smokers	Non-smokers	−0.705 (0.342)	2.025 (1.036 to 3.956)	0.039*
Constant			−1.474(0.310)		< 0.001

Model χ^2^ = 0.005, −2Log likelihood = 362.277, Cox & Snell R^2^ = 0.059, Nagelkerke R^2^ = 0.093, *p* = 0.708 (Hosmer and Lemeshow test)

## Discussion

Appropriate MMP is essential to ensure medication safety, and to minimize the possibility of overdosing and wastage of resources [15, 21]. However, the majority of the participants (80%) in our study had inappropriate home MMP. Our finding may be the indication of inadequate information provided by the healthcare professionals to such individuals in our setting, probably due to their limited knowledge [22, 23]. MMP is not always optimal or appropriate in both developing and developed countries. In Uganda, 70% of the study participants had inappropriate MMP at home [5]. Similarly, the MMP among Australian [4, 6, 17] and American [18] population has also been noted unsatisfactory. Chronically ill individuals face numerous difficulties on a day-to-day basis for the management of their own medications and it is challenging to develop, maintain, and adjust their medication routines [24]. Moreover, a study in a different setting has found low health literacy levels among patients with chronic diseases [25], and inappropriate medication management has been linked with low literacy level since such individuals are unable to read and understand the medication instruction [26]. Nearly three-fifths of participants had never gone to school, therefore, it might also be responsible for comparatively higher inappropriate practice as seen in our study. In addition to this, inadequate pharmaceutical service delivery by hospital pharmacy [27, 28] and delivery of service by unauthorized personnel in the community pharmacy of Nepal [29] might also have contributed to the findings of our study.

In bivariate analysis of our study, the MMP (appropriate or inappropriate) was found to be associated only with duration of disease (*p* = 0.002), which indicated that chances of inappropriate MMP increases with increase in the duration of disease. However, multivariate analysis showed a significant association between MMP with both the duration of disease and smoking status. This analysis showed that individuals aged ≥60 years with a lesser duration of the disease had higher odds of appropriate MMP but the odds were statistically significant only in participants with disease duration of less than one year and 1–2 years when compared with the duration of more than three years. Alternatively, longer duration of the disease can be considered as a predictor of inappropriate MMP at home. This highlights the importance of assessment of knowledge of older patients on medication management on follow-up visits and continuing education in order to improve their practice and therapeutic outcomes. A study in Uganda depicts that on multivariate analysis longer disease duration is significantly associated with inappropriate home MMP. The likelihood of inappropriate MMP was double among those with more than five years disease duration than those with five or less years [5]. Though this study was not specifically on older patients and undertook patients with treatment for chronic disease for at least two months; it suggested that patients with prolonged diseases may be associated with drug accumulation, visiting multiple prescribers, and perceiving their disease as very severe, thus leading to poor MMP.

Similarly, in our study smokers had two times lesser odds of appropriate home MMP than non-smokers. A possible explanation for this finding is that smoking is found to be a significant risk factor for cognitive decline and dementia among older adults [30–32]. Therefore, non-smokers might have a good cognition leading to better home MMP in our study. However, the effect of smoking on MMP in people aged above 60 years needs further elaboration. A study from Uganda states that perceived severity of disease, duration of disease of more than five years, and lack of treatment-response assessment by health workers as predictors of inappropriate medication practice [5]. Another study from Australia showed greater number of medication at home as predictor of therapeutic duplication, hoarding and greater severity of illness [6].

It has been known that patients with threshold or sub-threshold health literacy have lower ability to perform simple tasks and actualize the importance of tests or medicines [26]. Therefore, it necessitates the improvement of the level of health literacy of chronically ill older population which could be possible through interventions [33, 34] in order to improve MMP at home. The current status among those with poor literacy levels might also be improved through pictorial and audio-visual demonstrations. Moreover, older age has been known to be associated with decrease in memory power [35–37]. Furthermore, cognitive impairment has been observed in a number of diseases [38–42]. These indications emphasize the necessity of continuous education and counselling on aspects of medication management for chronically ill older individuals, though cognitively impaired patients might need dedicated attention.

Our study was a population-based study and we evaluated the medications of our participants at their homes, which signifies its strength. We were able to physically evaluate their medications as they would bring them out on every occasion, though we had to rely on verbal information from the participants about medication storage. However, the direct evaluation enabled us to obtain valid information about their medicines irrespective of their storage place. This highly reduced the recall bias or possibility of misinformation.

The sites of our study were selected on the basis of our convenience due to constraints of finance and manpower. Hence, probability sampling techniques such as stratified or cluster methods can be considered. Study population from rural areas and health services-deprived areas could make this study more inclusive. Additionally, this study could have incorporated more medication-related components such as dose and use of any alternative medicines. Furthermore, we collected information on all medicines that were available, but in the analysis, we excluded OTC and prn medicines. But we recommend considering the issue of self-medication practice of prescription medicine, particularly of antibiotics, in future studies in similar settings, as more recent finding suggests that antibiotics are dispensed without prescription by non-pharmacists from randomly selected community pharmacies in two districts of Nepal [43]. Moreover, whilst storing medicines in a cool and dry place (unless otherwise stated for different storage condition), out of the reach of children or avoiding potentially humid places such as bathrooms or kitchens, is an appropriate MMP, realistically achieving these practice in these group of patients in similar settings is seemingly difficult. However, an effort has to be directed towards improvising such practice. Similarly, consequences of medicine hoarding should be explained to patients and latter should be encouraged to return unused portions of discontinued medication to the pharmacy. This can be best achieved by advising them to bring all of their medications during their visit to the hospital or community pharmacy and by performing medication reconciliation.

## Conclusions

The home MMP among chronically ill older population in the selected areas of Nepal was poor. Duration of disease and smoking status were the predictors of medication management by older population at home in our study. It is possible to improve MMP of older patients by early intervention. Healthcare professionals including physicians, pharmacists and nurses should all re-assess and adequately counsel the older patients about MMP at home during their health institutions visits.

## Additional file

Additional file 1: Data collection tool. A sheet designed to collect information on four different aspects of participants including socio-demographic characteristics, illness characteristics, life styles and medication management practice. (PDF 301 kb)

## Abbreviations

CI: Confidence interval
IQR: Interquartile range
MMP: Medication management practice
OR: Odds ratio
SPSS: Statistical package for social sciences
