# Supplementary material for: Factors predicting home medication management practices among chronically ill older population of selected districts of Nepal
Source: BMC Geriatr. 2019 Feb 27;19:55. doi: 10.1186/s12877-019-1081-7 (PMC6391813; doi:10.1186/s12877-019-1081-7)
Supplement: Supplementary file 1 — Data collection tool. A sheet designed to collect information on four different aspects of participants including socio-demographic characteristics, illness characteristics, life styles and medication management practice. (PDF 301 kb) [file 12877_2019_1081_MOESM1_ESM.pdf]

DATA COLLECTION TOOL

Section 1: Socio-demographic information of patients

1) Age: …………
2) Sex: a. Male: ☐ b. Female: ☐
3) Education:
   a. Never went school: ☐ c. Secondary: ☐ e. ≥Graduation: ☐
   b. Primary: ☐ d. Higher-secondary: ☐
4) Occupation:
   a. Unemployed: ☐ b. Government job: ☐
   c. Private job: ☐ d. Business: ☐
5) Marital status:
   a. Unmarried: ☐ c. Divorced: ☐ e. Separated: ☐
   b. Married: ☐ d. Widowed: ☐

Section 2: Illness characteristics

6) Type of diseases
   a. Asthma: ☐ c. Hypertension: ☐ e. Rheumatic Arthritis: ☐
   b. Diabetes: ☐ d. Heart diseases: ☐ f. other:___________
7) Duration of diseases
   a. More than 3 months ☐ b. More than 6 months ☐
   b. More than 9 months ☐ c. More than 1 year ☐
8) Symptoms ____
9) Severity of diseases
   a. Mild☐ b. Moderate☐ c. Severe☐

Section 3: Life styles

10) Do you take oily/spicy food? a. Yes☐ b. No☐
11) Do you take alcohol? a. Yes☐ b. No☐
12) Do you smoke? a. Yes☐ b. No☐
13) Do you do exercise? a. Yes☐ b. No☐

Section 4: Medication Management

14) Have medication administration schedule? a. Yes☐ b. No☐
15) Presence of medication hoarding that were discontinued? a. Yes☐ b. No☐
16) Presence of expired medicine(s)? a. Yes☐ b. No☐
17) Had multiple prescribers for a disease condition? a. Yes☐ b. No☐
18) Was the route of administration appropriate: a. Yes☐ b. No☐
19) Storage condition of medicines?
   a. Appropriate ☐
   b. Inappropriate☐ (Please tick why inappropriate)
      ☐no specific location ☐multiple areas
      ☐wrong container ☐inappropriate location
20) Is there any medication duplication? a. Yes☐ b. No☐
